# Progress and prospects in research and clinical practice of hormone receptor-positive, HER-2-negative breast cancer with *BRCA1*/2 mutations

**DOI:** 10.1007/s12672-023-00732-0

**Published:** 2023-06-23

**Authors:** Shunchao Yan, Murshid Imam

**Affiliations:** Department of Oncology, Shengjing Hospital of China Medical University, Shenyang, 110022 China

**Keywords:** Breast cancer, BRCA mutation, Cancer treatment, PARP inhibitor, Hormone receptor-positive

## Abstract

Breast cancer (BC) is a heterogeneous disease that is the most common cancer in women worldwide. However, precise subtyping and corresponding treatments have improved patient outcomes. Hormone receptor (HR)-positive, human epidermal growth factor receptor type 2 (HER2)-negative (HR+/HER2-) BC with BRCA1 and/or BRCA2 mutations (BRCA1/2m) is a unique BC subset with dual drivers: homologous recombination deficiency and hormone receptor signaling. Wild-type BRCA1/2 suppresses estrogen receptor-mediated signaling. Loss-of-function mutations in *BRCA1*/2 release estrogen receptor suppression, leading to reduced sensitivity to endocrine therapy. Poly (ADP-ribose) polymerase (PARP) inhibitors (PARPis) exert antitumor effects against this subtype and can be used in combination with endocrine therapy. Although PARPis have been evaluated in metastatic triple-negative breast cancer, their efficacy against HR+/HER2- BC has not been clearly established. The present review summarizes recent advances and prospects in the progress of the HR+/HER2-/BRCA1/2m subgroup. As such, this article provides theoretical guidance for future research and promotes the use of PARPis for the treatment of HR+/HER2-/BRCA1/2m BC.

## Introduction

Breast cancer (BC) is a heterogeneous disease that became the most common female cancer in 2020 [1]. Over the past 20 years, considerable research has been conducted on the molecular subtyping of BC [2]. The disease is classified into distinct molecular subtypes according to the expression of estrogen receptor (ER), progesterone receptor (PR), human epidermal growth factor receptor type 2 (HER2), and Ki67 [3]. Precision therapy for molecular subtypes has substantially improved outcomes of patients with BC [4]. However, more than 40,000 women die of BC every year in the United States alone and 500,000 deaths occur worldwide [5, 6]. Consequently, there is an ongoing need to develop novel therapeutic strategies.

Mutations in the BC susceptibility genes *BRCA1* and *BRCA2* (BRACA1/2m) increase the risk of developing BC [7]. Loss-of-function mutations in germline *BRCA1* and/or *BRCA2* (gBRCA1/2m) occur in > 5% of unselected BC patients and ~ 30% of patients with a family history of BC [8, 9]. The hormone receptor-positive (HR+) subtype accounts for approximately 70% of all BC cases, while ~ 2–8% of HR + BC cases harbor gBRCA1/2m [10, 11]. In the HR low positive group (1–9% ER and/or PR), BRCA1/2m is even more prevalent (approximately 40%) [12]. BRCA1 and BRCA2 are essential for homologous recombination repair of DNA double-strand breaks [13] and have myriad functions in cell survival, growth, and division [14, 15]. Cancers carrying gBRCA1/2m are characterized by homologous recombination DNA repair deficiency [16]. This results in selective sensitivity to poly (ADP-ribose) polymerase (PARP) inhibitors (PARPis), which inhibit and trap the DNA repair enzyme PARP via synthetic lethality [17]. The OlympiAD and EMBRACA trials provided evidence that PARPi treatment is superior to chemotherapy in terms of efficacy and toxicity in HER2-negative (HER2-) metastatic BC (mBC) [18, 19]. Thus, talazoparib and olaparib have been approved for the treatment of HER2- mBC with BRCAm.

HR+/HER2-/BRCA1/2m BC is a unique BC subtype with dual drivers: homologous recombination deficiency and hormone receptor signaling. Although PARPis have been broadly considered in metastatic triple-negative BC (TNBC), their efficacy in the treatment of HR+/HER2- mBC remains to be fully established. There is still some confusion regarding the topological optimization of the treatment among oncologists and unmet medical needs for patients. This review summarizes recent advances in the treatment of patients with HR+/HER2- BC in the BRCA1/2m subgroup. In addition, we highlight current challenges and strategies that provide theoretical guidance for future research on managing this subgroup.

## BRCAm status in HR + BC

### BRCAm frequency and function

BRCA1 protein contains a RING finger domain in the N-terminus with E3 ubiquitin ligase activity. Meanwhile, the C-terminus domain of BRCA1 participates in DNA break repair and contains numerous phosphoprotein binding sites. BRCA2 contains a transactivation domain in the N-terminus and a long RAD51-specific binding domain and DNA binding domain toward the C-terminus [20–22]. It contributes to DNA repair by regulating the RAD51 protein [23].

Hundreds of mutations have been identified in BRCA1/2, including nonsense mutations, deletions, and insertions, most of which result in functionally inactive proteins [17]. Loss-of-function (LOF) mutations are widely scattered across *BRAC1* and *BRCA2*, affecting gene structure and function. Given that the functions of different mutation sites vary, they can result in different domain truncated or null proteins. Most LOF mutations are localized in the BRCA1/2 C-terminus core or the transcription factor binding sites, thus impacting transcriptional activation by *BRCA1/2* [22, 24]. Given that BRCA1/2 participates in cell division by regulating various molecular events during mitosis, mutations that cause functional transcriptional disruption of *BRCA1/2* may lead to cancer development. These mutations can disrupt cell signal-regulated processes, leading to disease progression and drug resistance.

The *BRCA1* and BRCA2 gene mutation sites also vary based on patient ethnicity, likely due to differences in genetic background [25]. Mutations in *BRCA1/2* are associated with most early-onset hereditary BC cases [26]. In particular, LOF mutations in *BRCA1* and/or *BRCA2* confer a high lifetime risk of developing BC (45–80%) by age 70 [27]. Individuals carrying a *gBRCA1* mutation tend to develop TNBC [28, 29], whereas those carrying *gBRCA2* mutations are predisposed to develop HR + BC [30]. Approximately 5% of HR + BC patients and 13.7% of TNBC patients carry BRCAm [27–29]. More specifically, the rates of mutation in HR+/Her-2- BC are 1.7% for BRCA1 and 3.3% for BRCA2; whereas in TNBC, the mutation rate is 12.6% for BRCA1 and 1.1% for BRCA2 [11]. Meanwhile, somatic mutations in BRCA1 rarely occur in unselected patients [9, 31–33]. BRCA1 expression typically decreases in sporadic BC, thereby enhancing sporadic BC progression [32, 33]. However, to date, no study has reported whether the hot-spot BRCA1/2 mutations in HR + HER2- BC differ from other BC subtypes, thus warranting additional investigation.

### Genetic testing status of BRCAm

As genetic testing was not widely available worldwide prior to the last decade, most patients with BC without a family history have not been tested for BRCAm status [34, 35]. Testing rates vary widely among different molecular subtypes of BC [36, 37]. Previously, doctor recommendations for *BRCA* gene testing tended to favor patients with TNBC or those with a family history of BC or ovarian cancer. Therefore, the frequencies of BRCAm have been underestimated in patients with HR+/HER2-BC. Meanwhile, approximately 50% of all BCs with BRCA1/2m are of the HR+/HER2 subtype [38, 39]. PARPis were approved by the Food and Drug Administration (FDA) and the European Medicines Agency for the treatment of HER2- advanced BC with BRCA1/2m in 2018. Since then, the frequency of BRCAm testing has increased [40]. A recent study assessed the rate of BRCA1/2m testing in HER2- advanced BC in the United States, Israel, and Europe from October 2019 to March 2020 [41]. The rates of gBRCA1/2m testing are still relatively low among the HR+/HER2- subgroups, although BRCA1/2m testing has increased. In the United States, 93% of TNBC cases have been assayed for BRCAm status, while only 68% of HR+/HER2- cases are tested. In addition, the rate of BRCA1/2m testing in HR+/HER2- advanced BC decreases with age. In Europe, 78% of TNBC cases are tested for BRCA status, while only 37% of HR+/HER2- BC patients are tested. This is likely lower in developing countries [42].

Potential obstacles to BRCAm testing for HR+/HER2- BC include insufficient understanding of the importance of BRCAm for the treatment of this molecular subtype, cost of testing, and patient perception toward BRCA testing [43–45]. The European Society for Medical Oncology and the National Comprehensive Cancer Network Clinical Practice Guidelines in Oncology (NCCN guidelines) recommend that patients with advanced or metastatic BC be tested for BRCA1/2m status. The cost-effectiveness of BRCA1/2m testing varies among countries [45], and the use of the same testing criteria is not feasible for all countries due to resource inconsistencies [46].

## Characteristics of patients with BRCAm + BC

### Epidemiological and clinicopathological characteristics

The estimated average cumulative risk of BC in individuals aged 70 years is approximately 52 and 47% for BRCA1 and BRCA2m carriers, respectively [47]. The risk of second primary contralateral BC is 83% in BC patients with BRCA1m and 62% in BC patients with BRCA2m [48]. The pathological type of BC with BRCA1/2m differs from sporadic and familial BC without BRCA1/2m. Meanwhile, the occurrence of invasive ductal/lobular carcinoma is not substantially different between carriers of BRCA1/2m and sporadic BC patients. However, atypical medullary or medullary carcinomas arise more often in BRCA1 carriers (13%) than in BRCA2m carriers (3%) or sporadic BC patients (2%) [49]. BC with gBRCAm typically exhibit more aggressive behavior, higher Ki67 expression, and more lymph node metastases than BC patients without BRCA1/2m [13, 50, 51]. Moreover, gBRCA1/2m HR + BC is more likely to be diagnosed at a younger age (< 45 years) than sporadic HR + BC [52, 53]. gBRCAm HR + BC is also associated with a higher nuclear grade than sporadic HR + BC. In fact, gBRCAm HR + BC patients have a ~ 3-fold increased rate of high recurrence compared with the general BC population [54–56]. Unlike BCs without BRCAm, HR positivity has no favorable prognostic value in patients with BRCA-mutated BC; this population represents a high-recurrence risk subgroup [57]. In addition, patients with a BRCA1m are more often diagnosed as p53-positive with a higher tumor grade. Meanwhile, BC with BRCA2m has a degree of malignancy between those of sporadic and BC with BRCA1m [58].

### BRCAm and outcomes in HR + BC

The prognostic value of BRCA1/2m in BC remains controversial. Some studies suggest that patients with BC and BRCA1/2m experience a higher risk of distant recurrence and BC-related death than sporadic/BRCA- individuals [59, 60]. However, other studies have shown that patients with BC and BRCA1/2m have better overall survival (OS) than the general BC population [61–63]. A meta-analysis investigated the oncological safety of breast-conserving surgery therapy in BRCAm carriers. These carriers had a significantly higher risk of ipsilateral BC recurrence than non-carriers, with a median follow-up of ≥ 7 years [64]. Moreover, the risk of contralateral BC (CBC) in BC patients with gBRCAm is greater than in non-carriers [65]. A high gene mutation load is associated with poor survival in patients with HR + BC, while defects in DNA damage repair (DDR) genes, such as *BRCA*, may be drivers of endocrine therapy (ET) resistance in HR + BC [66]. In addition, HR+/HER2- mBC with BRCAm is less sensitive to CDK4/6 inhibitors than the HR+/HER2- BRCA wild-type (WT) phenotype [67].

## BRCAm and cell signals in HR + BC

### BRCAm impact on HR protein expression and estrogen synthesis

**Fig. 1:**
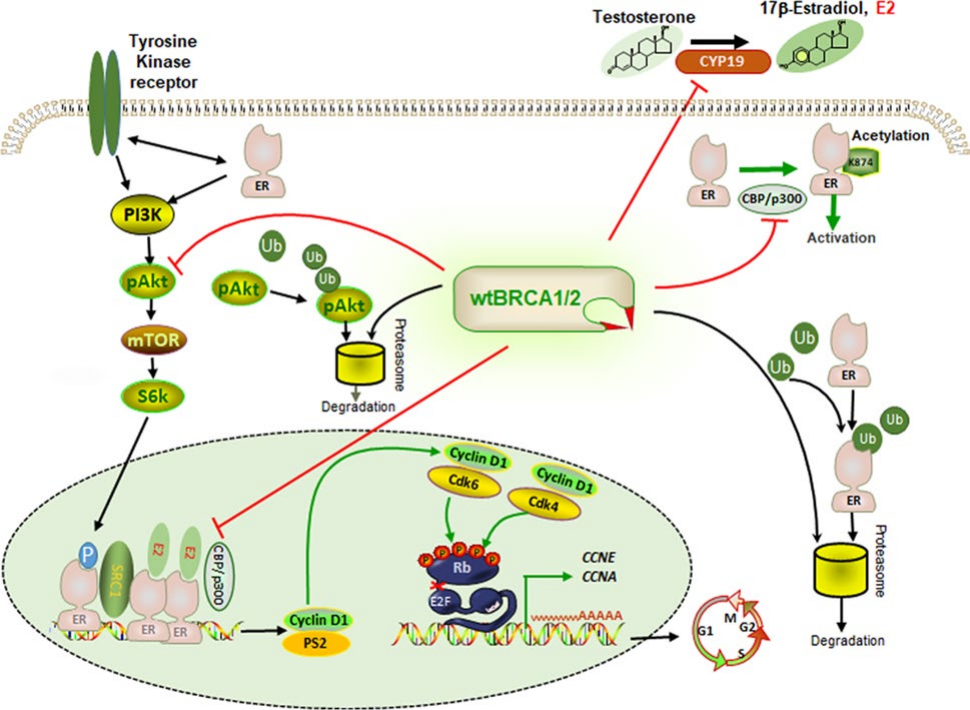
Wild-type BRCA1/2 regulating the expression of estrogen receptor (ER), production of estrogen, and genomic and non-genomic pathways of ER.

BRCA is involved in the regulation of HR status and activity (Fig. 1). The E3 ubiquitin ligase activity of the BRCA1 N-terminal RING finger domain elicits tumor suppressor functions, which can be enhanced by heterodimerization with the BARD1 protein. ER is a ubiquitination substrate for BRCA1/BARD1 ubiquitin ligase, which recognizes and monoubiquitinates the ligand-binding domain of ER [68]. Mutations in the N-terminal RING finger domain of BRCA1 abrogate the inhibition of ER activity [69]. PR is also degraded through the proteasome pathway, which is mediated by BRCA1/BARD1 [70, 71]. King et al. suggested that BRCA1/2m increases PR expression [72].

In premenopausal women, systemic estrogen is synthesized by granulosa cells in the ovaries. In postmenopausal women, plasma estrogen is produced depending on the key enzyme, aromatase, in other organs. In vitro, WT BRCA1 negatively regulates aromatase expression, thereby decreasing the concentration of plasma estrogen and further suppressing ER-α activity [73, 74]. Consistently, aromatase expression is upregulated in BRCA1m carriers [75], and plasma estrogen concentrations are elevated by up to 30% [76]. Therefore, WT BRCA1 inhibits ER-α-driven signaling, and BRCA1m can release this inhibition. Moreover, a retrospective study explored the chemoprevention effect of aromatase inhibitor (AI) treatment in non-metastatic ER + BC patients who carry BRCA mutations but do not undergo contralateral prophylactic mastectomy. Adjuvant AI therapy reduced the risk of contralateral BC in BRCA mutation carriers [77]. Hence, HR + BRCAm patients represent a subpopulation that is at a higher risk and whose disease physiology requires further characterization. Nevertheless, this subgroup benefits from adjuvant aromatase inhibitor therapy.

### BRCAm and estrogen signaling pathway

Acetylation is a post-translational modification important for the physiological activity of proteins. P300 directly interacts with and acetylates the ER-α lysine motif, which is necessary for the transcriptional activation of the ER. BRCA1 inhibits p300 expression and indirectly inhibits ER-α acetylation. Conversely, BRCA1m results in hyperacetylation and increased transcriptional activity of the ER [69, 78]. DDR defects are drivers of endocrine treatment resistance [79]. Estrogen directly stimulates BC growth through ER genomic and estrogen-independent non-genomic pathways. In vitro studies have demonstrated that the amino-terminal region of WT BRCA1 can physically interact with the conserved carboxyl terminal activation (AF-2) of ER, which inhibits the activity of ligand-activated ER [80]. The LOF of BRCA1m abolishes the capacity to inhibit ER-α genomic pathway activity. The PI3K/AKT/mTOR pathway is an important non-genomic ER signaling pathway. BRCA1 contains phosphoprotein binding domains, which can interact with p-AKT, leading to its ubiquitination and degradation. BRCA1 deficiency or mutation increases the kinase activity of AKT [81].

In addition, activated ER signaling promotes the cell cycle by upregulating the expression of cyclin D1 [82]. Overactivation of the cyclin D-CDK4/6–Rb pathway is also a critical inducer of resistance to ET. Currently, CDK 4/6 inhibitors are the first-line target treatment for HR + mBC [83]. BC patients with BRCA1/2m typically express higher levels of cyclin D1 than patients without BRCA1/2m, as evidenced by their clinical samples [84]. Overexpressed Cyclin D1 results in CDK4/6 inhibitor resistance in BC cells. Downregulation of Cyclin D1 leads to cell cycle arrest in the G1 phase and restores sensitivity to CDK4/6 inhibitors [85].

## Clinical development of PARPi in HR+/HER-2- BC

### PARPi in neoadjuvant and adjuvant treatment in HR+/HER-2- early BC patients with BRCAm

The phase II GeparOLA study compared neoadjuvant olaparib and paclitaxel with carboplatin and paclitaxel in HER2- BC patients with homologous recombinant deficiency. In the ER+/HER2- subgroup, patients administered olaparib + paclitaxel treatment achieved a pathologic complete response (pCR) of 52.6%, while those administered carboplatin + paclitaxel achieved only 20% pCR. In addition, HR+/HER2- BC patients with gBRCA1/2m administered olaparib + paclitaxel achieved 67% (10/15 patients) pCR, whereas no BRCA1/2 WT HR+/HER2- BC patients (0/4 patients) achieved pCR, demonstrating that PARPis exhibited marked antitumor effects in this subgroup [86]. The Neotala study, a non-randomized phase II study on BRCA-mutated tumors, investigated the efficiency of neoadjuvant talazoparib alone. The subgroup analysis showed that 60% of ER+/HER2- patients achieved pCR, higher than that of the total population (53%) [87]. The evidence for using PARPis in neoadjuvant therapy of ER+/HER2- BC with BRCA1/2m is insufficient, and more phase III clinical trials are warranted in this regard.

Olympia was a large phase III study that investigated the efficacy of intensive adjuvant therapy with olaparib for one year after traditional adjuvant therapy in HER2- early BC patients carrying gBRCA1/2m. The CPS + EG score combines pretreatment clinical and posttreatment pathologic stage (CPS), ER status (E), and grade (G) to estimate the risk of recurrence. The ER+/HER2- BC patients included in the study had high-risk factors for recurrence (non-pCR and a CPS + EG score ≥ 3 in patients who received neoadjuvant therapy, or ≥ 4 positive lymph nodes after initial surgery). Olaparib treatment increased 3-year invasive disease-free survival by 19% in ER+/HER2- BC patients who received neoadjuvant chemotherapy, although the difference was not significant (86 vs. 67%, hazard ratio 0.52, 95% confidence interval 0.25–1.04), likely due to the small patient population (*N* = 196, 11% of all patients) [88]. In June 2021, these data prompted the update of the NCCN guidelines that now recommended 1 year of intensive adjuvant therapy with olaparib for ER+/HER2- early BC with BRCA1/2m and residual disease and a CPS + EG score ≥ 3 in the case of previous neoadjuvant chemotherapy or ≥ 4 positive lymph nodes after the initial surgery. However, the Olympia trial included HR+/HER2-/gBRCAm early BC patients with a high risk of recurrence while excluding those with low or medium recurrence risks who may have also benefited from PARPi treatment. Further clinical trials on expanding the range of individuals who can benefit from PARPi treatment warrant careful study design.

### PARPis in HR+/HER-2- mBC with BRCA1/2m

Several PARPis have been extensively studied for the treatment of gBRCAm carriers with metastatic HER2- BC and have demonstrated the efficiency of PAPRis in the HR + and TNBC subgroups (Table 1). Olaparib was approved by the FDA based on the OlympiAD trial in January 2018 [18, 89]. Another PARPi, talazoparib, was approved by the FDA based on EMBRACA trials in October 2018 [19]. The OlympiAD and EMBRACA trials compared single-agent PARPi to non-platinum single-agent chemotherapy for gBRCAm mBC. In the OlympiAD trial, HR+/HER2- patients received at least one prior ET for metastatic disease. In the subgroup analysis, compared to chemotherapy, olaparib did not improve progression-free survival (PFS) in ER+/HER2- mBC patients but achieved a good objective response rate (65.4%). However, in the EMBRACA study, talazoparib considerably improved PFS in patients with ER+/HER2- mBC [90]. Furthermore, compared with chemotherapy, PARPi treatment improved health-related quality of life in the OlympiAD and EMBRACA trials. However, neither study reported improvements in the OS with PARPi treatment. Meanwhile, in the BROCADE3 trial, HER2- BC patients with gBRCAm were treated with veliparib and chemotherapy, followed by maintenance treatment with veliparib, and compared against HER2- BC patients who were subjected to chemotherapy and a placebo. Veliparib considerably improved PFS in TNBC and ER+/HER2- mBC patients [91] but did not improve OS in any of the subgroups. The LUCY trial, an observational prospective real-world study, evaluated the effectiveness of olaparib in gBRCAm, TNBC, and HR + BC [92]. The authors found that patients with TNBC and HR + BC subjected to olaparib treatment achieved consistent PFS. These studies provide considerable evidence for the application of PARPis in HR+/HER2- mBC patients with gBRCA1/2m.

**Table 1:** Characteristics of studies reporting the efficiency of PARP inhibitors in HR+/HER2-/BRCAm mBC

Clinical trial	PARP inhibitor	Patient population	Treatment arms and results of all randomized patients	Treatment arms and results of the HR+/HER2- subgroup	Treatment arms and results of the TNBC subgroup
OlympiAD (NCT02000622)	Olaparib	≤ 2 previous cytotoxic regimens for advanced BC. Previous taxane and/or anthracycline. DFI > 12 months after platinum treatment. No limit of previous endocrine therapy, unless one prior ET.	Arm 1: Olapair, N = 205. Arm 2: Standard therapy, N = 97. HR for PFS = 0.58 (0.43–0.80), *p* < 0.001 HR for OS = 0.90 (0.63–1.29), *p* = NS ORR: 59.9% vs. 28.8%	Arm 1: Olapair, N = 103. Arm 2: Standard therapy, N = 49. HR for PFS = 0.82 (0.55–1.26), *p* = NS HR for OS = 0.86 (0.55–1.36), *p* = NS ORR: 65.4% vs. 36.4%	Arm 1: Olapair, N = 102. Arm 2: Standard therapy, N = 49. HR for PFS = 0.43 (0.29–0.63), *p* = NA HR for OS = 0.93 (0.62–1.43), *p* = NS ORR: 54.7% vs. 21.2%
EMBRACA (NCT01945775)	Talazoparib	≤ 3 previous cytotoxic regimens for advanced BC. Previous taxane and/or anthracycline. DFI > 6 months after platinum treatment. No limit of previous endocrine ET.	Arm 1: Talazoparib, N = 287. Arm 2: Overall PCT, N = 144. HR for PFS = 0.54 (0.41–0.71), *p* < 0.0001 HR for OS = 0.848 (0.670–1.073), *p* = NS ORR: 62.6% vs. 27.2%	Arm 1: Talazoparib, N = 157. Arm 2: Overall PCT, N = 84. HR for PFS = 0.47 (0.32–0.71), *p* = 0.0002 HR for OS = 0.827 (0.597–1.143), *p* = NS ORR: 63.2% vs. 37.9%	Arm 1: Talazoparib, N = 130. Arm 2: Overall PCT, N = 60. HR for PFS = 0.60 (0.41–0.87), *p* = 0.0075 HR for OS = 0.899 (0.634–1.276), *p* = NS ORR: 61.8% vs. 12.5%
BROCADE3 (NCT02163694)	Veliparib	≤ 2 previous cytotoxic regimens for advanced breast cancer. Previous taxane allowed but given > 6 or 12 months before the start of the study in (neo)adjuvant or metastatic setting, respectively. DFI > 12 months after platinum treatment. No limit of previous ET.	Arm 1: Veliparib plus C/P, N = 337. Arm 2: Placebo plus C/P, N = 172. HR for PFS = 0.82 (0.63–1.07), *p* = NS HR for OS = NA ORR: 75.8% vs. 74.1%	Arm 1: Veliparib plus C/P, N = 174. Arm 2: Placebo plus C/P, N = 92. HR for PFS = 0.69 (0.52–0.93), *p* = 0.013 HR for OS = 0.96 (0.68–1.36), *p* = NS ORR: 73.9% vs. 71.1%	Arm 1: Veliparib plus C/P, N = 163. Arm 2: Placebo plus C/P, N = 80. HR for PFS = 0.72 (0.52–1.00), *p* = 0.052 HR for OS = 0.92 (0.65–1.31), *p* = NS ORR: 77.6% vs. 77.6%
LUCY (NCT03286842)	Olaparib	≤ 2 previous cytotoxic regimens for advanced breast cancer. Previous taxane and/or anthracycline. DFI > 12 months after platinum treatment. No limit of previous endocrine therapy, unless one prior ET.	Arm 1: Olaparib, N = 252. mPFS: 8.11 months Arm 2: NA (No comparison, single arm)	Arm 1: Olaparib, N = 131. mPFS = 8.34 months Arm 2: NA	Arm 1: Olaparib, N = 121. mPFS = 6.8 months Arm 2: NA

*BC* breast cancer, *DFI* disease-free interval, *PFS* progression-free survival, *OS* overall survival, *HR* hazard ratio, *ET* endocrine therapy, *PCT* physician’s choice of chemotherapy, *C/P* carboplatin/paclitaxel, *NA* not available, *NS* not significant, *ORR* objective response rate

However, these studies compared the efficiency of PARPi with chemotherapy or placebo, not ET-based therapy (including ET plus CDK4/6 inhibitor), which is the first choice for HR+/HER2- mBC patients without visceral crises. Therefore, PARPi treatment should be considered after ET-based treatment and before chemotherapy in these patients. Meanwhile, a recent phase I/II trial explored the safety and efficacy of a combined CDK4/6 inhibitor, olaparib, and ET in mBC [93]; however, the potential adverse effects require further discussion. Moreover, additional clinical trials are needed to compare the efficiency of ET + PARPi vs. ET + CDK4/6 inhibitor in HR+/HER-2- mBC with BRCA1/2m.

## Clinical outcomes of CDK4/6 inhibitors in BRCAm HR+/HER2- patients

ET plus CDK4/6 inhibitors have been strongly recommended for the treatment of HR+/HER2- mBC patients as the first and subsequent lines [94]. The results of the MonarchE study support the administration of abemaciclib (2 years) to patients with high-risk HR+/HER2- early BC as an intensive adjuvant therapy on the basis of ET [95]. However, there is no evidence supporting the administration of ribociclib or palbociclib in the adjuvant treatment of early BC. No data has been published regarding the efficiency of CDK4/6 inhibitors in early BRCAm HR+/HER2- patients.

Additionally, the registered trials for CDK4/6 inhibitors lack pre-specified subgroup analysis plans for gBRCA1/2m carriers. In the MonaLEEsa-2, − 3, and − 7 studies, the patients with HR+/HER2- mBC were treated with ET alone or ribociclib + ET as the first- or second-line treatment [96–98]. A pooled biomarker analysis of these trials showed that BRCA1/2m was a potential biomarker of sensitivity to ribociclib-based treatment, that is, the PFS of BRCA1/2m patients was relatively improved compared with BRCA1/2 wild patients (*P* > 0.05 but considered actionable) [99]. However, other studies have shown opposing results. For instance, in the PADA-1 trial, patients with HR+/HER2- mBC were administered an aromatase inhibitor plus palbociclib as the first-line treatment. A subsidiary analysis of the PADA-1 trial showed that gBRCAm or gPALB2m patients tended to have a shorter PFS than gBRCAwt patients (14.3 m versus 26.7 m; HR 0.58 (95% CI [0.2–1.02]), *P* = 0.056) [100]. Meanwhile, a real-world study retrospectively analyzed the association between the mutation of DNA repair-related genes and the efficacy of CDK 4/6 inhibitor plus ET in patients with HR+/HER2- advanced BC. The results showed that gBRCA1/2-ATM-CHEK2 pathogenic variants were independently associated with poor outcomes [101]. Collins, et al. further extracted data for 2968 patients from the Flatiron Health database with HR+/HER2-mBC treated with a CDK4/6 inhibitor and analyzed clinical outcomes based on gBRCA status in a real-world setting. The results showed that patients with gBRCAm had a significantly shorter OS time (sHR 1.50; 95% CI 1.06–2.14) than those with gBRCAwt after CDK4/6 inhibitor treatment [67]. In an MSKCC cohort analysis, gBRCA2 mutations predicted worse PFS (HR 2.17, 95% CI 1.46–3.22, *P* < 0.001) for first-line therapy with ET plus CDK4/6 inhibitor in HR+/HER2- mBC patients [102]. Hence, CDK4/6i plus ET treatment may be less effective for patients with gBRCAm than in those without BRCAm, although the current results are conflicting. Moreover, BRCAm HR+/HER2- BC patients might benefit from the early introduction of a PARP inhibitor. Taken together, these results demonstrate an unmet need for the treatment of patients with HR+/HER2- and gBRCAm. Accordingly, prospective studies are warranted to assess the efficiency of CDK4/6 inhibitors in HR+/HER2- mBC with BRCA1/2m.

## Conclusions and perspectives

HR+/HER2- BC with BRCA1/2m is a special subset of BC. BRCA1/2m releases the inhibition of ER signaling and introduces PARPi into the treatment regimen of this BC subtype. This review provides an overview of recent advances in the field of HR + and BRCA1/2m in BC and discusses approaches and directions for future clinical trials. More attention should be paid to determining BRCA1/2m status in patients with HR+/HER2- early BC and mBC. For HR+/HER2- early BC with BRCA1/2m with high-risk factors for recurrence, olaparib + ET and CDK4/6 inhibitor + ET represent two potential options for postoperative intensive adjuvant therapy. The question of which is the best option has been debated since the results of the Olympia study were published. Some oncologists have explored the effect of sequential administration of CDK4/6 inhibitors and olaparib for intensive adjuvant therapy to avoid under-inhibition of cell proliferation in early BC but without supporting evidence. Moreover, for HR+/HER2- mBC with BRCA1/2m, PARPi treatment should be considered after CDK4/6 inhibitor treatment and before chemotherapy. No published data have compared PARPi + ET with CDK4/6i + ET in HR+/HER2- mBC with BRCA1/2m, which is a direction for further clinical trials. Additionally, the safety of combining a CDK4/6 inhibitor and a PARPi requires further verification. The benefits of PARPi treatment should be taken into consideration when making systemic treatment decisions; however, additional clinical trials are warranted to optimize the treatment schedule for CDK4/6 inhibitors and PARPis in HR+/HER2-/gBRCAm early and advanced BC.

## Data Availability

Data sharing is not applicable to this article as no datasets were generated or analyzed during the current study.

## References

[1] Siegel RL, Miller KD, Fuchs HE, Jemal A (2022). Cancer statistics, 2022. CA Cancer J Clin.

[2] Russnes HG, Lingjærde OC, Børresen-Dale A-L, Caldas C (2017). Breast cancer molecular stratification: from intrinsic subtypes to integrative clusters. Am J Pathol.

[3] Al-Thoubaity FK (2020). Molecular classification of breast cancer: a retrospective cohort study. Ann Med Surgery.

[4] Gu G, Dustin D, Fuqua SA (2016). Targeted therapy for breast cancer and molecular mechanisms of resistance to treatment. Curr Opin Pharm.

[5] Giaquinto AN, Sung H, Miller KD, Kramer JL, Newman LA, Minihan A (2022). Breast cancer statistics, 2022. CA Cancer J Clin.

[6] Torre LA, Bray F, Siegel RL, Ferlay J, Lortet-Tieulent J, Jemal A (2015). Global cancer statistics, 2012. CA Cancer J Clin.

[7] Li JY, Jing R, Wei H, Wang M, Xiaowei Q, Liu H (2019). Germline mutations in 40 cancer susceptibility genes among C hinese patients with high hereditary risk breast cancer. Int J Cancer.

[8] Godet I, Gilkes DM (2017). BRCA1 and BRCA2 mutations and treatment strategies for breast cancer. Integr cancer Sci Ther.

[9] Winter C, Nilsson MP, Olsson E, George AM, Chen Y, Kvist A (2016). Targeted sequencing of BRCA1 and BRCA2 across a large unselected breast cancer cohort suggests that one-third of mutations are somatic. Ann Oncol.

[10] Timms KM, Abkevich V, Hughes E, Neff C, Reid J, Morris B (2014). Association of BRCA1/2 defects with genomic scores predictive of DNA damage repair deficiency among breast cancer subtypes. Breast Cancer Res.

[11] Tung N, Lin NU, Kidd J, Allen BA, Singh N, Wenstrup RJ (2016). Frequency of germline mutations in 25 cancer susceptibility genes in a sequential series of patients with breast cancer. J Clin Oncol.

[12] Sanford RA, Song J, Gutierrez-Barrera AM, Profato J, Woodson A, Litton JK (2015). High incidence of germline BRCA mutation in patients with ER low‐positive/PR low‐positive/HER‐2 neu negative tumors. Cancer.

[13] Venkitaraman AR (2001). Functions of BRCA1 and BRCA2 in the biological response to DNA damage. J Cell Sci.

[14] Bertwistle D, Ashworth A (1998). Functions of the BRCA1 and BRCA2 genes. Curr Opin Genet Dev.

[15] Yoshida K, Miki Y (2004). Role of BRCA1 and BRCA2 as regulators of DNA repair, transcription, and cell cycle in response to DNA damage. Cancer Sci.

[16] O’Kane GM, Connor AA, Gallinger S (2017). Characterization, detection, and treatment approaches for homologous recombination deficiency in cancer. Trends Mol Med.

[17] Lord CJ, Ashworth A (2017). PARP inhibitors: synthetic lethality in the clinic. Science.

[18] Robson M, Tung N, Conte P, Im S-A, Senkus E, Xu B (2019). OlympiAD final overall survival and tolerability results: Olaparib versus chemotherapy treatment of physician’s choice in patients with a germline BRCA mutation and HER2-negative metastatic breast cancer. Ann Oncol.

[19] Litton J, Hurvitz S, Mina L, Rugo H, Lee K-H, Gonçalves A (2020). Talazoparib versus chemotherapy in patients with germline BRCA1/2-mutated HER2-negative advanced breast cancer: final overall survival results from the EMBRACA trial. Ann Oncol.

[20] Zhang F, Ma J, Wu J, Ye L, Cai H, Xia B (2009). PALB2 links BRCA1 and BRCA2 in the DNA-damage response. Curr Biol.

[21] Yang H, Jeffrey PD, Miller J, Kinnucan E, Sun Y, Thoma NH (2002). BRCA2 function in DNA binding and recombination from a BRCA2-DSS1-ssDNA structure. Science.

[22] Murphy CG, Moynahan ME (2010). BRCA gene structure and function in tumor suppression: a repair-centric perspective. Cancer J.

[23] Thorslund T, West S (2007). BRCA2: a universal recombinase regulator. Oncogene.

[24] Mirkovic N, Marti-Renom MA, Weber BL, Sali A, Monteiro AN (2004). Structure-based assessment of missense mutations in human BRCA1: implications for breast and ovarian cancer predisposition. Cancer Res.

[25] Ou J, Wu T, Sijmons R, Ni D, Xu W, Upur H (2013). Prevalence of BRCA1 and BRCA2 germline mutations in breast cancer women of multiple ethnic region in northwest China. J Breast Cancer.

[26] Dite GS, Jenkins MA, Southey MC, Hocking JS, Giles GG, McCredie MR (2003). Familial risks, early-onset breast cancer, and BRCA1 and BRCA2 germline mutations. J Natl Cancer Inst.

[27] Triantafyllidou O, Vlachos IS, Apostolou P, Konstantopoulou I, Grivas A, Panopoulos C (2015). Epidemiological and clinicopathological characteristics of BRCA-positive and BRCA-negative breast cancer patients in Greece. J BUON.

[28] Lakhani SR, Van De Vijver MJ, Jacquemier J, Anderson TJ, Osin PP, McGuffog L (2002). The pathology of familial breast cancer: predictive value of immunohistochemical markers estrogen receptor, progesterone receptor, HER-2, and p53 in patients with mutations in BRCA1 and BRCA2. J Clin Oncol.

[29] Atchley DP, Albarracin CT, Lopez A, Valero V, Amos CI, Gonzalez-Angulo AM (2008). Clinical and pathologic characteristics of patients with BRCA-positive and BRCA-negative breast cancer. J Clin Oncol.

[30] Mavaddat N, Barrowdale D, Andrulis IL, Domchek SM, Eccles D, Nevanlinna H (2012). Pathology of breast and ovarian cancers among BRCA1 and BRCA2 mutation carriers: results from the Consortium of investigators of modifiers of BRCA1/2 (CIMBA) Tumor Pathology in BRCA1 and BRCA2 mutation carriers. Cancer Epidemiol Biomarkers Prev.

[31] Khoo U-S, Ozcelik H, Cheung AN, Chow LW, Ngan H, Done SJ (1999). Somatic mutations in the BRCA1 gene in chinese sporadic breast and ovarian cancer. Oncogene.

[32] Sourvinos G, Spandidos DA (1998). DecreasedBRCA1Expression levels may arrest the cell cycle through activation ofp53Checkpoint in human sporadic breast tumors. Biochem Biophys Res Commun.

[33] Thompson ME, Jensen RA, Obermiller PS, Page DL, Holt JT (1995). Decreased expression of BRCA1 accelerates growth and is often present during sporadic breast cancer progression. Nat Genet.

[34] Rajagopal PS, Nielsen S, Olopade OI (2019). USPSTF recommendations for BRCA1 and BRCA2 testing in the context of a transformative national cancer control plan. JAMA Netw Open.

[35] Hull LE, Haas JS, Simon SR (2018). Provider discussions of genetic tests with US women at risk for a BRCA mutation. Am J Prev Med.

[36] Lux MP, Lewis K, Rider A, Niyazov A (2020). Abstract P6–08-09: BRCA1/2 testing in adult women with HER2-advanced breast cancer (ABC): results from a US real world study. Cancer Res.

[37] Murphy A, Elit L, Bell K, Pond G, Bordeleau L (2017). Referral rate for, and uptake of genetic testing in women diagnosed with breast cancer ≤ 35 and triple negative breast cancer (TNBC) ≤ 50. Ann Breast Cancer Ther.

[38] Collet L, Péron J, Penault-Llorca F, Pujol P, Lopez J, Freyer G (2022). PARP inhibitors: a major therapeutic option in endocrine-receptor positive breast cancers. Cancers.

[39] Li J, Wen WX, Eklund M, Kvist A, Eriksson M, Christensen HN (2019). Prevalence of BRCA1 and BRCA2 pathogenic variants in a large, unselected breast cancer cohort. Int J Cancer.

[40] Cardoso F, Paluch-Shimon S, Senkus E, Curigliano G, Aapro M, André F (2020). 5th ESO-ESMO international consensus guidelines for advanced breast cancer (ABC 5). Ann Oncol.

[41] Mahtani R, Niyazov A, Arondekar B, Lewis K, Rider A, Massey L (2022). BRCA1/2 mutation testing in patients with HER2-Negative advanced breast cancer: real-world data from the United States, Europe, and Israel. Cancers.

[42] Krivokuca A, Mihajlovic M, Susnjar S, Spasojevic IB, Minic I, Popovic L (2022). Mutational profile of hereditary breast and ovarian cancer–establishing genetic testing guidelines in a developing country. Curr Probl Cancer.

[43] Stuckey A, Febbraro T, Laprise J, Wilbur JS, Lopes V, Robison K (2016). Adherence patterns to national comprehensive cancer network guidelines for referral of women with breast cancer to genetics professionals. Am J Clin Oncol.

[44] Pal T, Cragun D, Lewis C, Doty A, Rodriguez M, Radford C (2013). A statewide survey of practitioners to assess knowledge and clinical practices regarding hereditary breast and ovarian cancer. Genet Test Mol Biomarkers.

[45] Forbes C, Fayter D, de Kock S, Quek RG (2019). A systematic review of international guidelines and recommendations for the genetic screening, diagnosis, genetic counseling, and treatment of BRCA-mutated breast cancer. Cancer Manag Res.

[46] Alemar B, Gregorio C, Herzog J, Matzenbacher Bittar C, Brinckmann Oliveira Netto C, Artigalas O (2017). BRCA1 and BRCA2 mutational profile and prevalence in hereditary breast and ovarian cancer (HBOC) probands from Southern Brazil: are international testing criteria appropriate for this specific population?. PLoS ONE.

[47] Milne RL, Osorio A, Cajal TRny, Vega A, Llort G, De La Hoya M (2008). The average cumulative risks of breast and ovarian cancer for carriers of mutations in BRCA1 and BRCA2 attending genetic counseling units in Spain. Clin Cancer Res.

[48] Molina-Montes E, Pérez-Nevot B, Pollán M, Sánchez-Cantalejo E, Espín J, Sánchez M-J (2014). Cumulative risk of second primary contralateral breast cancer in BRCA1/BRCA2 mutation carriers with a first breast cancer: a systematic review and meta-analysis. Breast.

[49] Stratton MR (1997). Pathology of familial breast cancer: differences between breast cancers in carriers of BRCA1 or BRCA2 mutations and sporadic cases. Lancet.

[50] Tredan O, De Talhouet S, Peron J, Friedlaender A, Vuilleumier A, Viassolo V (2018). Abstract P3–03-05: Association between BRCA1 and BRCA2 mutations and survival in breast cancer patients according to molecular subtype. Cancer Res.

[51] Aleskandarany M, Caracappa D, Nolan CC, Macmillan RD, Ellis IO, Rakha EA (2015). DNA damage response markers are differentially expressed in BRCA-mutated breast cancers. Breast Cancer Res Treat.

[52] Fountzilas E, Konstantopoulou I, Vagena A, Apostolou P, Papadimitriou C, Christodoulou C (2020). Pathology of BRCA1-and BRCA2-associated breast cancers: known and less known connections. Clin Breast Cancer.

[53] Krammer J, Pinker-Domenig K, Robson ME, Gönen M, Bernard-Davila B, Morris EA (2017). Breast cancer detection and tumor characteristics in BRCA1 and BRCA2 mutation carriers. Breast Cancer Res Treat.

[54] Shah PD, Patil S, Dickler MN, Offit K, Hudis CA, Robson ME (2016). Twenty-one–gene recurrence score assay in BRCA‐associated versus sporadic breast cancers: differences based on germline mutation status. Cancer.

[55] Halpern N, Sonnenblick A, Uziely B, Divinsky L, Goldberg Y, Hamburger T (2017). Oncotype Dx recurrence score among BRCA1/2 germline mutation carriers with hormone receptors positive breast cancer. Int J Cancer.

[56] Lewin R, Sulkes A, Shochat T, Tsoref D, Rizel S, Liebermann N (2016). Oncotype-DX recurrence score distribution in breast cancer patients with BRCA1/2 mutations. Breast Cancer Res Treat.

[57] Lambertini M, Ceppi M, Hamy A-S, Caron O, Poorvu PD, Carrasco E (2021). Clinical behavior and outcomes of breast cancer in young women with germline BRCA pathogenic variants. NPJ breast cancer.

[58] Song Y, Barry WT, Seah DS, Tung NM, Garber JE, Lin NU (2020). Patterns of recurrence and metastasis in BRCA1/BRCA2-associated breast cancers. Cancer.

[59] Baretta Z, Mocellin S, Goldin E, Olopade OI, Huo D (2016). Effect of BRCA germline mutations on breast cancer prognosis: a systematic review and meta-analysis. Medicine.

[60] Jin TY, Park KS, Nam SE, Yoo YB, Park WS, Yun IJ (2022). BRCA1/2 serves as a biomarker for poor prognosis in breast carcinoma. Int J Mol Sci.

[61] Cortesi L, Masini C, Cirilli C, Medici V, Marchi I, Cavazzini G (2010). Favourable ten-year overall survival in a caucasian population with high probability of hereditary breast cancer. BMC Cancer.

[62] Kriege M, Seynaeve C, Meijers-Heijboer H, Collee JM, Menke-Pluymers M, Bartels C (2009). Sensitivity to first-line chemotherapy for metastatic breast cancer in BRCA1 and BRCA2 mutation carriers. J Clin Oncol.

[63] Maksimenko J, Irmejs A, Nakazawa–Miklasevica M, Melbarde–Gorkusa I, Trofimovics G, Gardovskis J (2014). Prognostic role of BRCA1 mutation in patients with triple–negative breast cancer. Oncol Lett.

[64] Valachis A, Nearchou AD, Lind P (2014). Surgical management of breast cancer in BRCA-mutation carriers: a systematic review and meta-analysis. Breast Cancer Res Treat.

[65] Kriege M, Jager A, Hooning MJ, Huijskens E, Blom J, van Deurzen CH (2012). The efficacy of taxane chemotherapy for metastatic breast cancer in BRCA1 and BRCA2 mutation carriers. Cancer.

[66] Haricharan S, Bainbridge MN, Scheet P, Brown PH (2014). Somatic mutation load of estrogen receptor-positive breast tumors predicts overall survival: an analysis of genome sequence data. Breast Cancer Res Treat.

[67] Collins JM, Nordstrom BL, McLaurin KK, Dalvi TB, McCutcheon SC, Bennett JC (2021). A real-world evidence study of CDK4/6 inhibitor treatment patterns and outcomes in metastatic breast cancer by germline BRCA mutation status. Oncol Therapy.

[68] Eakin CM, MacCoss MJ, Finney GL, Klevit RE (2007). Estrogen receptor α is a putative substrate for the BRCA1 ubiquitin ligase. Proc Natl Acad of Sci.

[69] Ma Y, Fan S, Hu C, Meng Q, Fuqua SA, Pestell RG (2010). BRCA1 regulates acetylation and ubiquitination of estrogen receptor-α. Mol Endocrinol.

[70] Poole AJ, Li Y, Kim Y, Lin S-CJ, Lee W-H, Lee EY-H (2006). Prevention of Brca1-mediated mammary tumorigenesis in mice by a progesterone antagonist. Science.

[71] Calvo V, Beato M (2011). BRCA1 counteracts progesterone action by ubiquitination leading to progesterone receptor degradation and epigenetic silencing of target promoters. Cancer Res.

[72] King TA, Gemignani ML, Li W, Giri DD, Panageas KS, Bogomolniy F (2004). Increased progesterone receptor expression in benign epithelium of BRCA1-related breast cancers. Cancer Res.

[73] Lu M, Chen D, Lin Z, Reierstad S, Trauernicht AM, Boyer TG (2006). BRCA1 negatively regulates the cancer-associated aromatase promoters I. 3 and II in breast adipose fibroblasts and malignant epithelial cells. J Clin Endocrinol Metab.

[74] Ghosh S, Lu Y, Katz A, Hu Y, Li R (2007). Tumor suppressor BRCA1 inhibits a breast cancer-associated promoter of the aromatase gene (CYP19) in human adipose stromal cells. Am J Physiol-Endocrinol Metab.

[75] Chand AL, Simpson ER, Clyne CD (2009). Aromatase expression is increased in BRCA1mutation carriers. BMC Cancer.

[76] Widschwendter M, Rosenthal AN, Philpott S, Rizzuto I, Fraser L, Hayward J (2013). The sex hormone system in carriers of BRCA1/2 mutations: a case-control study. Lancet Oncol.

[77] Nemati Shafaee M, Goutsouliak K, Lin H, Bevers TB, Gutierrez-Barrera A, Bondy M (2022). Aromatase inhibitors and contralateral breast cancer in BRCA mutation carriers. Breast Cancer Res Treat.

[78] Fan S, Ma YX, Wang C, Yuan R-Q, Meng Q, Wang J-A (2002). p300 modulates the BRCA1 inhibition of estrogen receptor activity. Cancer Res.

[79] Anurag M, Punturi N, Hoog J, Bainbridge MN, Ellis MJ, Haricharan S (2018). Comprehensive profiling of DNA repair defects in breast cancer identifies a novel class of endocrine therapy resistance drivers. Clin Cancer Res.

[80] Fan S, Wang J-A, Yuan R, Ma Y, Meng Q, Erdos M (1999). BRCA1 inhibition of estrogen receptor signaling in transfected cells. Science.

[81] Xiang T, Ohashi A, Huang Y, Pandita TK, Ludwig T, Powell SN (2008). Negative regulation of AKT activation by BRCA1. Cancer Res.

[82] Eeckhoute J, Carroll JS, Geistlinger TR, Torres-Arzayus MI, Brown M (2006). A cell-type-specific transcriptional network required for estrogen regulation of cyclin D1 and cell cycle progression in breast cancer. Genes Dev.

[83] Kwapisz D (2017). Cyclin-dependent kinase 4/6 inhibitors in breast cancer: palbociclib, ribociclib, and abemaciclib. Breast Cancer Res Treat.

[84] Aaltonen K, Blomqvist C, Amini R-M, Eerola H, Aittomäki K, Heikkilä P (2008). Familial breast cancers without mutations in BRCA1 or BRCA2 have low cyclin E and high cyclin D1 in contrast to cancers in BRCA mutation carriers. Clin Cancer Res.

[85] Cai Z, Wang J, Li Y, Shi Q, Jin L, Li S (2023). Overexpressed cyclin D1 and CDK4 proteins are responsible for the resistance to CDK4/6 inhibitor in breast cancer that can be reversed by PI3K/mTOR inhibitors. Sci China Life Sci.

[86] Fasching P, Link T, Hauke J, Seither F, Jackisch C, Klare P (2021). Neoadjuvant paclitaxel/olaparib in comparison to paclitaxel/carboplatinum in patients with HER2-negative breast cancer and homologous recombination deficiency (GeparOLA study). Ann Oncol.

[87] Telli ML, Litton JK, Beck JT, Jones JM, Andersen J, Mina LA (2021). Neoadjuvant talazoparib (TALA) in patients (pts) with germline BRCA1/2 (g BRCA1/2) mutation-positive, early HER2-negative breast cancer (BC): exploration of tumor BRCA mutational status and zygosity and overall mutational landscape in a phase 2 study. Wolters Kluwer Health.

[88] Geyer C, Garber J, Gelber R, Yothers G, Taboada M, Ross L (2022). Overall survival in the OlympiA phase III trial of adjuvant olaparib in patients with germline pathogenic variants in BRCA1/2 and high-risk, early breast cancer. Ann Oncol.

[89] Robson M, Im S-A, Senkus E, Xu B, Domchek SM, Masuda N (2017). Olaparib for metastatic breast cancer in patients with a germline BRCA mutation. New Engl J Med.

[90] Rugo HS, Ettl J, Hurvitz SA, Gonçalves A, Lee K-H, Fehrenbacher L (2020). Outcomes in clinically relevant patient subgroups from the EMBRACA study: talazoparib vs physician’s choice standard-of-care chemotherapy. JNCI cancer spectrum.

[91] Diéras V, Han HS, Kaufman B, Wildiers H, Friedlander M, Ayoub J-P (2020). Veliparib with carboplatin and paclitaxel in BRCA-mutated advanced breast cancer (BROCADE3): a randomised, double-blind, placebo-controlled, phase 3 trial. Lancet Oncol.

[92] Gelmon KA, Fasching PA, Couch FJ, Balmaña J, Delaloge S, Labidi-Galy I (2021). Clinical effectiveness of olaparib monotherapy in germline BRCA-mutated, HER2-negative metastatic breast cancer in a real-world setting: phase IIIb LUCY interim analysis. Eur J Cancer.

[93] Torres A, Kokkonen C, Oladeji M, D’Andrea K, Mick R, Narayan V (2022). Abstract OT2-18-01: Harnessing olaparib, palbociclib, and endocrine therapy (HOPE): Phase I/II trial of olaparib, palbociclib and fulvestrant in patients with BRCA1/2-associated, hormone receptor-positive, HER2-negative metastatic breast cancer. Cancer Res.

[94] Wu Y, Zhang Y, Pi H, Sheng Y (2020). Current therapeutic progress of CDK4/6 inhibitors in breast cancer. Cancer Manag Res.

[95] Harbeck N, Rastogi P, Martin M, Tolaney SM, Shao ZM, Fasching PA (2021). Adjuvant abemaciclib combined with endocrine therapy for high-risk early breast cancer: updated efficacy and Ki-67 analysis from the monarchE study. Ann Oncol.

[96] Hortobagyi G, Stemmer S, Burris H, Yap Y, Sonke G, Hart L (2021). LBA17 overall survival (OS) results from the phase III MONALEESA-2 (ML-2) trial of postmenopausal patients (pts) with hormone receptor positive/human epidermal growth factor receptor 2 negative (HR+/HER2–) advanced breast cancer (ABC) treated with endocrine therapy (ET) ± ribociclib (RIB). Ann Oncol.

[97] Slamon D, Neven P, Chia S, Fasching P, De Laurentiis M, Im S-A (2019). Overall survival (OS) results of the phase III MONALEESA-3 trial of postmenopausal patients (pts) with hormone receptor-positive (HR+), human epidermal growth factor 2-negative (HER2–) advanced breast cancer (ABC) treated with fulvestrant (FUL) ± ribociclib (RIB). Ann Oncol.

[98] Tripathy D, Im S-A, Colleoni M, Franke F, Bardia A, Harbeck N (2018). Ribociclib plus endocrine therapy for premenopausal women with hormone-receptor-positive, advanced breast cancer (MONALEESA-7): a randomised phase 3 trial. Lancet Oncol.

[99] Andre F, Su F, Solovieff N, Arteaga CL, Hortobagyi GN, Chia SK (2020). Pooled ctDNA analysis of the MONALEESA (ML) phase III advanced breast cancer (ABC) trials. Am Soc Clin Oncol.

[100] Frenel J, Dalenc F, Pistilli B, Rouge TdLM, Levy C, Mouret-Reynier M (2020). 304P ESR1 mutations and outcomes in BRCA1/2 or PALB2 germline mutation carriers receiving first line aromatase inhibitor + palbociclib (AI + P) for metastatic breast cancer (MBC) in the PADA-1 trial. Ann Oncol.

[101] Bruno L, Ostinelli A, Waisberg F, Enrico D, Ponce C, Rivero S (2022). Cyclin-dependent kinase 4/6 inhibitor outcomes in patients with advanced breast cancer carrying germline pathogenic variants in DNA repair–related genes. JCO Precision Oncol.

[102] Safonov A, Bandlamudi C, de Lara PT, Ferraro E, Derakhshan F, Will M (2022). Abstract GS4-08: comprehensive genomic profiling of patients with breast cancer identifies germline-somatic interactions mediating therapy resistance. Cancer Res.

